# Policies for healthy and sustainable edible oil consumption: a stakeholder analysis for Thailand

**DOI:** 10.1017/S1368980016003037

**Published:** 2016-11-24

**Authors:** Bhavani Shankar, Nalitra Thaiprasert, Shabbir Gheewala, Richard Smith

**Affiliations:** 1 Leverhulme Centre for Integrative Research in Agriculture and Health and Centre for Development, Environment and Policy, SOAS University of London, 36 Gordon Square, London WC1H 0PD, UK; 2 Faculty of Economics, Chiang Mai University, Chiang Mai, Thailand; 3 Joint Graduate School of Energy and Environment, Centre of Excellence on Energy Technology and Environment, King Mongkut’s University of Technology Thonburi (KMUTT), Bangkok, Thailand; 4 Faculty of Public Health & Policy, London School of Hygiene & Tropical Medicine, London, UK

**Keywords:** Edible oil, Saturated fat, Policy, CVD, Stakeholder analysis

## Abstract

**Objective:**

Palm oil is a cheap and versatile edible oil in widespread use as a food ingredient that has been linked to negative health and environmental outcomes. The current study aimed to understand the prospects for future health-focused policy development to limit food use of palm oil and promote a greater diversity of oils in Thailand’s food system.

**Design:**

Eighteen semi-structured interviews were conducted with a range of stakeholders. The interviews probed views on the economic, health and environmental dimensions of the issue, the prospects for health-focused policy development and the policy development process. Transcripts were analysed using a health policy analytical framework.

**Setting:**

Thailand.

**Subjects:**

Stakeholders from a range of ministries, regulatory agencies, the private sector, non-governmental organizations and academia.

**Results:**

There are several impediments to the emergence of strong regulation, including the primacy of economic considerations in setting policy, doubt and misperception about health implications and a complex regulatory environment with little space for health-related considerations. At the same time, some sections of the food industry producing food for domestic consumption are substituting palm with other oils on the basis of consumer health perceptions.

**Conclusions:**

Strong regulation to curb the growth of palm oil is unlikely to emerge soon. However, a long-term strategy can be envisaged that relies on greater policy support for other indigenous oils, strategic rebalancing towards the use of palm oil for biofuels and oleochemicals, and harnessing Thailand’s food technology capabilities to promote substitution in food production in favour of oils with healthier fatty acid composition.

Chronic disease accounts for over 60 % of deaths globally, with low- and middle-income countries now accounting for nearly 80 % of the disease burden associated with non-communicable diseases (NCD) such as CVD^(^ ^1^ ^)^. Nutrition transitions have been implicated as a key driver of the increased prevalence of NCD, particularly CVD, in low- and middle-income countries^(^ ^2^ ^)^. These transitions typically involve a move away from diets rich in complex carbohydrates and fibre towards diets with greater proportions of fats, saturated fats and sugar^(^ ^3^ ^,^ ^4^ ^)^. The replacement of unsaturated fats and/or high-quality carbohydrates with saturated fats in diets is linked to higher risk of CHD^(^ ^5^ ^)^. Reflecting the importance of the composition of fats, the Global Monitoring Framework for NCDs includes proportions of energy intake from saturated and polyunsaturated fatty acids in its indicator list^(^ ^6^ ^)^.

The transition towards diets high in saturated fats in low- and middle-income countries is frequently marked by a rapid expansion in edible oil consumption^(^ ^7^ ^)^. Edible oils have contributed more to the global increase in energy availability since the 1980s than any other food group. The rise of palm oil, driven by its low cost of production and versatility as a food ingredient, has been particularly spectacular. Globally, the use of vegetable oil has increased by a factor of 4·5 times since 1980, but palm oil consumption has increased by more than 10 times during this period^(^ ^8^ ^)^. Palm oil has experienced the fastest growth among all vegetable oils during the last three decades and now accounts for a 33 % share in global vegetable oil consumption^(^ ^8^ ^)^. A major reason for palm oil’s rapid spread is that oil palm yields substantially more oil per hectare than competing oilseeds^(^ ^9^ ^)^, resulting in a major cost advantage. Although biofuel use has contributed to the rise of palm oil, the bulk of global palm oil consumption continues to be as food^(^ ^10^ ^)^. Apart from its use as a cooking oil, palm oil is an ingredient in a large variety of processed food products, ranging from baked and fried goods to instant noodles and coffee whiteners. The major producers of palm oil have a significant economic stake in the sector, with palm oil contributing significantly to their exports, employment and Gross Domestic Product. For example, palm oil accounts for 5–6 % to Malaysia’s of Gross Domestic Product and 62 % of its value of exports^(^ ^11^ ^)^.

The widespread substitution of palm for other oils has health implications, since palm oil is relatively high in saturated fat content (on average, approximately 50 %) and relatively low in polyunsaturated fat content (on average, approximately 10 %) compared with oils that it has displaced, such as soyabean and sunflower oils. A recent systematic review^(^ ^12^ ^)^ found that palm oil raises LDL cholesterol in comparison to vegetable oils with lower saturated fat, supporting the notion that it is desirable to substitute palm oil with oils with superior fatty acid profiles in diets. The environment is another important dimension to consider in any strategic thinking about the edible oil sector. Oil palm agricultural expansion has been linked to tropical deforestation^(^ ^13^ ^)^, which, particularly in Malaysia and Indonesia, has been shown to be a major cause of greenhouse gas emissions and species extinction^(^ ^14^ ^)^.

Thus there is a case for many countries to consider a movement from the current palm oil-dominated status quo towards the incorporation of a greater variety of edible oils in their food systems. Government policy will have a critical role to play in any such strategic movement since the edible oil sector, and particularly palm oil, in such countries is influenced by a variety of policy measures, including retail price controls and subsidies, import controls, oil production incentives, agricultural zoning regulations, etc. Thus a broad strategy would incorporate health considerations into traditional agricultural and food policy design^(^ ^15^ ^,^ ^16^ ^)^. Examples of policies introduced in other countries to limit saturated fat intake include Denmark’s tax on foods high in saturated fat content^(^ ^17^ ^)^ and Mauritius’ replacement of palm oil with soyabean oil in subsidized ration cooking oil supplies^(^ ^18^ ^)^. In the present paper we consider the prospects for future health (and environment)-sensitive policy making in the edible oil sector using a case study approach. We report the results of a stakeholder analysis undertaken in Thailand to examine opportunities and challenges of developing such policies, considering the context, content, process and actors.

Thailand is ideal as a case study since it is both a significant producer (2 million tonnes of production in 2013^(^ ^19^ ^)^) and consumer (1·4 million tonnes of domestic utilization in 2013^(^ ^19^ ^)^) of palm oil, is grappling with nutrition transition and a significant burden of NCD^(^ ^20^ ^)^, and is a food processing giant. CVD is among the top causes of disease burden in Thailand^(^ ^21^ ^)^, with IHD and cerebrovascular disease being the first and third ranked contributors to years of life lost in 2013^(^ ^22^ ^)^. The prevalence of dyslipidaemia in the Thai population is high^(^ ^23^ ^)^ and dietary risks constitute the leading risk factor for burden of disease^(^ ^22^ ^)^.

Removal of *trans*-fats in the form of partially hydrogenated vegetable oils from the food chain is an immediate priority given the strength of evidence on their negative health impacts. A difficulty in this is that highly saturated tropical oils such as palm and coconut are the oils that can most easily substitute for partially hydrogenated vegetable oils in food processing, since they provide comparable oxidizability, stability and texture to partially hydrogenated vegetable oils^(^ ^24^ ^)^. However, the largest health benefits arise from substituting *trans*- with *cis*-unsaturated fats^(^ ^25^ ^)^. Thus a key strategic, technological and policy challenge is to manage a transition in the food industry from *trans*-fats to unsaturated fats^(^ ^24^ ^)^. This is recognized by the Global Framework for Monitoring of NCDs that lists replacement of *trans*-fats with polyunsaturated fats as a ‘best buy’ for NCD prevention^(^ ^6^ ^)^. However, there are also potential health benefits to be gained by a general replacement of saturated fatty acids with polyunsaturated fats in the diet^(^ ^5^ ^)^. Palm oil is present throughout the Thai food system, as the predominant cooking oil, and in foods ranging from instant noodles to milk products.

The present study contributes to the currently small literature on stakeholder analysis of health-focused policy development in the oils and fats sector^(^ ^26^ ^–^ ^29^ ^)^. This literature’s predominant focus is on *trans*-fats. In comparison to the literature, the study’s focus on a highly saturated fat, palm oil, presents several points of interest. First, it is important to note that the evidence around the health effects of saturated fat generally, and palm oil particularly, is the subject of significant current debate^(^ ^12^ ^,^ ^30^ ^–^ ^32^ ^)^. Thus this is a contested space, in contrast to the clear-cut evidence on *trans*-fats^(^ ^30^ ^)^. Second, palm oil confers significant economic benefits on producing and consuming countries, and this is a major factor in shaping the strength of some stakeholder positions. Third, oil palm cultivation has been associated with strong environmental impacts elsewhere in South-East Asia. Thus there is potentially an additional impetus for regulation arising from environmental concerns. Fourth, palm oil acts as a substitute for *trans*-fats in some food processing applications. This complicates the health implications of change and any efforts to regulate each of these kinds of fats, because both have been associated with negative health implications, and regulation may induce substitution between these fats and thereby blunt prospects of health improvement. Fifth, palm oil also has significant biofuel use, particularly in Thailand, and this has implications for palm oil use in the food sector. The objective of the study was to understand the prospects for future health-focused policy development to limit palm oil expansion and promote a greater diversity of edible oils in the Thai food system, while taking into account these five specific points of interest.

## Methods

### Stakeholder interviews: sampling

We conducted semi-structured interviews of stakeholders in health-focussed policy change in the palm oil sector in Thailand in two rounds between February and April 2015. The ethics review board of the lead author’s institution reviewed and approved the ethical review application. An initial list of key stakeholders for the first round of interviews was drawn up based upon the research team’s knowledge of the edible oil sector in Thailand. We identified respondents with a significant stake in policy making related to the production, consumption and trade of palm and other oils, including health, economic and environmental aspects. Snowball sampling led to the addition of further interviewees covered in the second round until theoretical saturation was reached and no new suggestions emerged. Conducting the interviews in two rounds enabled us to: (i) take into account in the second round initial information emerging from the first round; and (ii) refine our stakeholder set and provide us with adequate lead time to schedule interviews with new stakeholders added to the set.

Eighteen stakeholders were interviewed overall, eight from the public sector, seven from the private sector and three from non-governmental organizations and academia. Each of the interviewees was a senior representative of their institution, often the Director or the Chief Executive Officer. Several government bodies in Thailand have a stake in the oil palm, palm oil and other edible oil sub-sectors, including the Food and Drug Administration of Thailand (FDA), the Bureau of Standards, and the Ministries of Commerce, Industry, Agriculture & Cooperatives, Health, and Natural Resources & the Environment. Representatives from all these bodies were interviewed. A nutritionist at the head of a health advocacy group and a clinical lecturer specialized in CVD provided further representation of the health sector. A petroleum producer that is an important user of palm oil as biodiesel, an institute providing environmental research services to industry and a food technologist with palm oil expertise were also interviewed.

Food industry interviewees included a producer of a range of foods that is a major user of edible oil and a large producer of potato chip products. Also included were edible oil value chain representatives including a regional oil palm farmer association, an industry association representing palm oil and oil palm producers, and individual firms producing palm oil and rice bran oil. Stakeholder representation specifically in rice bran oil production was chosen in addition to palm oil for the following reasons. First, the main oils in terms of production in Thailand are palm and palm kernel oil, soyabean oil and rice bran oil. However, much of the soyabean oil production is from imported soyabeans. Thus palm and rice bran oils are the most important indigenous oils, although palm oil is produced in much greater volumes. As discussed later in the paper, indigeneity is an important trait when considering substitutes to palm oil in this setting. Second, three stakeholders in the first round of interviews named rice bran oil producers as an important stakeholder constituency we should consider for our second round. Third, major recent success stories in substitution of palm oil in the Thai food industry have involved rice bran oil as the chosen alternative. Fourth, there is evidence to show that rice bran oil has potentially beneficial health effects beyond those arising from its fat composition^(^ ^25^ ^)^.

### Stakeholder interviews: interview procedures

An initial letter sent to each stakeholder explained the background and purpose of the project and stakeholder exercise and sought their consent for participation. A subsequent telephone call was made to confirm participation and arrange an interview appointment. Each interviewee signed an informed consent form prior to their interview. All interviews were conducted face-to-face by a team of three, consisting of two project collaborators and a research assistant. Permission to record interviews was requested in each case, and was granted in all cases but one. Interviewees responded in Thai or English, depending on their preference. Interviews typically lasted between 1 and 2 h. All interviews were transcribed verbatim, translated as necessary and cross-checked by two members of the team. Detailed written notes were taken in the one case where recording permission was not granted.

### Stakeholder interviews: content

All interviews contained a core of common questions seeking information on the key dimensions of the problem. This included the stakeholder’s view of importance of the broad issue being investigated; their perceived importance of various dimensions of the problem, namely economic, health and environmental; their perception of the existing policy framework and the need for change; their general support for health (and environment)-sensitive policy development in the edible oil sector and for specific policy directions, including intrusive (palm oil taxes, standards for saturated content of policies, changing land use) as well as non-intrusive (investing in new technologies, subsidies for alternative oils) policies; their view of the process by which policy in this area gets shaped in Thailand; and their view of the level of influence/power of themselves and others in influencing policy. Specific questions were also developed for each interviewee based upon their specialist knowledge. In keeping with the semi-structured interview design, the interviews accommodated considerable flexibility in allowing deviation from standard questions, with the pre-designed questions typically serving as points of departure for other lines of enquiry.

### Analysis of data

Walt and Gilson’s^(^ ^33^ ^)^ pioneering framework for health policy analysis was used as the analytical framework for the study. The Walt and Gilson framework organizes health policy analysis on the basis of four key elements: (i) the context that helps shape policy; (ii) the content of the policy; (iii) the actors, or the stakeholders, that influence and are influenced by policy; and (iv) the process of policy making, or the ways in which potential policies emerge on the agenda (or fail to gain traction) and are modified, formally introduced as policies and implemented. In analysing data, we followed procedures outlined by Ulin *et al.* ^(^ ^34^ ^)^ for analysis of qualitative data in public health. We read carefully through the interview transcripts, with information from the first phase of interviews informing the second phase. Interview transcripts were then coded manually. One broad layer of coding was deductive^(^ ^34^ ^)^, mapping blocks of interview text to the analytical framework categories (i.e. context, content, actors and process). Another layer of codes was inductive in nature, with codes based on broad themes emerging from the data. Inductive themes were mapped to the analytical framework categories, although in some cases the themes cut across framework categories. All coding was done by the principal investigator, who was present at all eighteen interviews.

## Results

Below, we present results organized on the basis of the analytical framework categories of context, content, actors and process. Themes identified by the thematic analysis are indicated in italic font and discussed within the most relevant category. Existing literature is used to triangulate and provide context to material from interviews where helpful.

### Context

Stakeholders from policy circles showed a good awareness of chronic disease issues pertinent to Thailand. However, among the entire set of interviewees, *doubt, misperception and conspiracy theories abounded about health implications* of palm and other edible oils. A majority of interviewees felt that there was insufficient, or insufficiently clear, evidence about the negative health effects of high palm oil intake to warrant punitive regulation on health grounds. Even some stakeholders who were generally supportive of policy to restrict saturated fat intake felt that more Thailand-specific trial evidence was needed. A health sector stakeholder noted:

‘In my opinion soybean or rice bran oil is healthier than palm oil, but we don’t have sufficient evidence to prove it. From a nutrigenomics perspective, the health of the Thai population may respond differently to oil intake than in Western populations. More evidence from Thailand is necessary.’

Some stakeholders noted that this is an area where misinformation and misperceptions abound. For example, more than one stakeholder noted a trend among some Thais to consume coconut oil, another highly saturated fat, on its own on a daily basis in the belief that it can provide health benefits such as digestive assistance and weight regulation. Such misperceptions may originate from commercial promotions based on insufficient evidence. For example, a recent review concluded that, despite claims on websites and in commercial literature, the available evidence does not support a link between increased coconut oil consumption and improved lipid profiles or reduced CVD risk^(^ ^35^ ^)^.

Another belief expressed by several stakeholders was that there may be a global conspiracy to demonize tropical oils such as palm while boosting the image of soyabean oil in which several countries in the West has a major stake. One respondent commented:

‘USA, the world’s largest soybean producer, will say palm oil is bad for health. But Malaysia, the world’s largest palm oil producer, will have a positive view of palm oil.’

On the oil palm production and environmental aspects, a key theme identified relating to context was that *oil palm cultivation in Thailand is not yet viewed as a major environmental threat*. Thailand’s experience and vision relating to oil palm cultivation is different from that of Malaysia and Indonesia, and most stakeholders were keen to emphasize this. First, even though oil palm is an important crop in Thailand, occupying about half a million hectares, Malaysia and Indonesia are much larger producers, with eight to ten times more area under oil palm than Thailand. Second, as many agri-food and environment sector interviewees pointed out, Thailand’s oil palm cultivation has been largely in the hands of small farmers rather than large plantations, and cultivation has been concentrated in areas formerly used for producing other crops. There is a strategic policy objective to expand oil palm area by 25 % by 2021, but this is expected to continue to be in former paddy fields, abandoned fruit orchards, wasteland, etc. Thus in terms of environmental impacts of palm, we often heard stakeholders note that ‘Thailand is not Malaysia or Indonesia’.

That said, little of Thai palm oil is currently certified by the Roundtable for Sustainable Palm Oil (RSPO). Explaining the reason for this, a palm oil industry stakeholder noted:

‘RSPO certification certainly creates additional costs. The challenge we face is in converting the broad criteria into practical options for the local farmers.’

The certification criteria that create additional costs include management practices such as chemical use and water requirements. Another industry stakeholder explained:

‘RSPO results in two types of costs: for upgrading, and for certification … when you have smallholder farmers operating one or two hectares, requiring a separate storage room for pesticides is costly … requiring buffer zones around farms to prevent contamination of water is costly.’

### Process

Palm oil is a commodity of substantial importance to the country given its multiple roles as a cooking oil, a food ingredient and a biofuel, and the small- and medium-scale farmer livelihoods that oil palm supports. Our interviews revealed a *complex regulatory environment that attempts to balance these multiple uses and objectives*. We summarize the process of palm oil policy formulation as revealed by our interviews below.

A National Palm Oil Policy Committee (NPOPC), chaired by the Deputy Prime Minister, is responsible for key policy decisions for the sector. The NPOPC consists of representatives of several ministries, quasi-governmental agencies, industry bodies, and oil palm and palm oil producers. However, Health is not represented on the Committee. Members discuss matters of key policy importance at NPOPC meetings, which serves as the basis for policy setting. The NPOPC has to balance multiple, often conflicting, objectives: to keep palm oil retail prices low, to offer competitive prices to oil palm producers, to meet biofuel demand, to prevent reliance on imports. An industry stakeholder described it as follows:

‘We are very helpful to each other … government, farmers, industry, refineries, talking to each other on a frequent basis … discussing questions such as “how should we prepare for the coming shortage in supply?”... “should we change the biofuel mandate?”’

While broader commodity-related policies specific to palm oil are set by NPOPC and are very important in determining palm oil consumption, a variety of direct food-related health policies can be envisaged that can influence intakes such as restrictions on saturated content of foods, labelling initiatives, etc. Such regulation falls under the purview of the FDA. The FDA consults academic experts and health foundations as needed in determining policy directions.

### Actors

Several interviewees identified the Ministries of Agriculture & Cooperatives and Commerce as the most influential among government departments in decision making relating to palm oil. Stakeholders in the palm oil supply chain are also well represented, with industry associations having a strong voice in NPOPC discussions. The FDA is the principal body charged with determining health-related food policy. The Ministry of Health and other actors in the health sector noted in our interviews that their role in this area is largely restricted to providing information and advice regarding nutrition and health aspects of food intake.

Our interviews revealed that *some sections of the food industry are already reformulating products by substituting other edible oils for palm oil. This is in spite of a lack of policy pressure to substitute oils, and arises due to consumer health perceptions*. The private sector has been developing opportunities in this area based upon healthy eating trends. Interviewees highlighted the prominent example of the largest potato chip maker in Thailand switching from palm oil to rice bran oil for its entire product line. This switch, informants noted, was due to the firm’s own assessment of an emerging preference among its target population for oils that are perceived to have superior health attributes. Subsequently, the second largest firm in this market has also developed a ‘healthier’ line of products that use rice bran oil in place of palm oil, and prominently advertises this aspect. An industry stakeholder noted that such reformulations were overturning many long-held beliefs and conventional wisdom among food technologists that certain food applications such as those that require deep frying would inevitably have to be based on highly saturated oils like palm oil.

Leading Thai edible oil producers are also developing and producing blends such as palm oil mixed with canola and camellia oil instead of pure palm oil. A leading producer of oils and fats in Thailand markets these blends of oils claiming they can ‘reduce the risk of heart disease and contain high vitamin E and omega 3, 6, 9’. Rice bran oil was highlighted by some of our interviewees as having particular potential for the future. Rice bran oil producers are trying to raise the profile of their oil, particularly among the middle and higher income classes in urban areas, by focusing on its antioxidant content and cholesterol-lowering properties. An interviewee who heads one of the top rice bran oil-producing firms in the world noted that the company had experienced very strong growth in recent years and was producing at maximum capacity. The interviewee described how the firm had deployed its team, including food technology and nutrition experts, to work with some major processed food producers in Thailand to incorporate rice bran oil in their products. Seemingly difficult concerns relating to oil suitability for deep frying, resultant product organoleptic properties, shelf-life, etc. had turned out to be unfounded or could be overcome. However, rice bran oil production does face its own challenges. The rice bran oil producer described how the supply of rice bran for oil extraction is strongly constrained due to on-farm quality control, infrastructural bottlenecks and inadequacies, and competition from animal feed use of rice bran. Rice bran oil receives no policy support. *Referring to palm oil’s status as a cheap edible oil consumed widely in Thailand, particularly by those on lower incomes*, a government stakeholder commented that:

‘Palm oil is supported because it is important for the poor. Rice bran and other such oils are for the rich’.

### Content

Although biofuel use is a key driver of growth in the sector, food use was highlighted as the most important use of palm oil by the policy stakeholders we interviewed, which is reflected in NPOPC’s policy making. The retail price of palm oil is capped to protect consumer economic interests, given how widespread the use of palm as cooking oil is. Soyabean oil is also subject to retail price control, but other edible oils are not. The subsidy structure contributes to palm oil usually being the cheapest edible oil in retail outlets (approximately 30 % cheaper than unsubsidized oils such as rice bran and sunflower oils at the time of our interviews). At the same time, the NPOPC aims to help oil palm and palm oil producers realize reasonable profits. A government stakeholder described the process of setting the retail price cap as ‘balancing the benefits between producers and consumers’. Imports are controlled and allowed only in particular circumstances, with only the Public Warehouse Corporation allowed to import palm oil. For example, when poor weather curtails domestic production, the Public Warehouse Corporation may allow limited imports.

Another equilibrating mechanism is the biofuel mandate, the requirement regarding the proportion of fuel that has to derive from biofuel sources. As a stakeholder from the petroleum industry put it:

‘The energy sector plays an important role in absorbing any excess palm oil production and acting as an internal price stabilizer.’

Currently the B7 mandate requires that diesel sold at the pump is blended with 7 % palm methyl ester (biodiesel). This may be revised by the NPOPC when excess or deficit production respectively threatens the stability of prices, profits and food use of palm oil. A stakeholder from the health sector observed that one approach to continue protecting the palm oil sector while taking health into consideration would be to reprioritize in the long run towards biodiesel and other oleochemical use of palm oil. Another stakeholder commented on the substantial potential for expansion in the use of Thai palm oil as an oleochemical in the production of a variety of cosmetics, personal care products and other applications. Several interviewees noted that any policy change specific to palm oil would have to be negotiated within the NPOPC, and that dominance of economic interests and the already complex regulatory environment limited the scope for introducing health-sensitive policy change.

With respect to oil palm production, considerable expansion is a stated goal for the future. A government stakeholder explained:

‘We have a plan for oil palm expansion over a 12 year period, 2015–2027. The objective of the plan is to ensure self-sufficiency for domestic consumption’.

The strategic objective is to expand oil palm cultivation in areas previously occupied by rice, rubber and abandoned orange orchards, preventing deforestation. Rubber producers are offered conversion payments to convert to oil palm.

With respect to the FDA’s policy making, *other food-related health policy priorities may take precedence* over issues relating to saturated fats. Our interviews revealed that FDA’s most pressing priorities include food safety, food additives and drug residues. Restriction of saturated fat intake is not incorporated in any existing policy measures, and even *trans-*fat regulation, often the highest priority area in health-related edible oil policy, is not yet on the horizon. As a stakeholder noted, the expense involved with passing regulation and most importantly, monitoring compliance, means that self-regulation by industry is the favoured approach.

Our interviews probed participant’s broad support for different types of policies, concerning both commodity policy relating to palm oil production, distribution and consumption, as well as health-related food policy such as fat taxes or restrictions on saturated fat content. Generally, we found that intrusive policies such as palm oil taxes did not find support among the majority of stakeholders. There was more support for less intrusive policies such as investing in new technologies.

## Discussion and conclusion

The current research has shown that a variety of factors combine to make it unlikely that strong regulation to curb the growth of palm oil in food use in Thailand will emerge in the medium term. First, economic considerations, and particularly the perceived need to provide cooking oil at low cost to poorer consumers, are paramount in this policy setting. Maintaining low prices for ‘basic’ foods has long been an objective and area of intervention for governments around the world, and may have political as well as economic underpinnings^(^ ^36^ ^)^. Second, health implications are not yet on the radar of those setting edible oil policy in Thailand. Furthermore, doubt and misperception about health implications present an obstacle to regulation, echoing findings from Mexico^(^ ^26^ ^)^, Costa Rica^(^ ^28^ ^)^ and India^(^ ^27^ ^)^ regarding lack of awareness of *trans*-fat health implications as a barrier to policy. The doubts expressed by stakeholders in Thailand about edible oils and saturated fat are understandable, particularly given that these issues are currently hotly contested even in the scientific arena^(^ ^12^ ^,^ ^30^ ^–^ ^32^ ^)^. Realistic policy recommendations will have to grapple with such widespread perception issues, expressed by a majority of our interviewees. Third, while potential environmental impacts can be an impetus for regulation in this area, Thailand’s palm oil production does not yet pose threats of deforestation to the degree that Malaysia and Indonesia have been experiencing for some decades. Furthermore, the currently limited scale of environmental impact from palm oil appears to contribute to a perception that impacts will continue to be limited in the future. Fourth, the complex regulatory system for palm oil already has to juggle several divergent interests and it is difficult to see additional health-related considerations entering in the medium term. Fifth, other areas such as food safety may take priority in health-related food policy making. This finding is consistent with Pérez-Ferrer *et al.* ^(^ ^26^ ^)^ who find in the context of policy making in Mexico that other competing nutrition agendas are prioritized over *trans*-fat regulation. Sixth, self-regulation and voluntary approaches are preferred when it comes to nutrition policy in Thailand, while strong regulation may be difficult to enact. ‘Traffic light’ nutrition labelling has been mooted and trialled in the past, but had to be dropped following protests by industry^(^ ^37^ ^)^. Since 2011, Guideline Daily Amounts labelling requirements have been introduced for five groups of snack products, but do not include saturated fat information^(^ ^37^ ^)^.

However, even if regulation that restricts palm oil is unlikely to emerge in the medium term, it is possible to envisage a long-term strategy promoting a greater diversity of oils in the system to capture potential health benefits. We sketch such a strategy below, comprising three main aspects that are interlinked and reinforce each other in achieving the main objective.

### 1. Greater support for indigenous ‘healthy’ oils

While regulation to curb palm oil may be politically challenging, assisting the development of alternative oils in the long run is less controversial or complex. Given our results show that there is suspicion among many stakeholders about the promotion of non-indigenous oils such as soyabean oil, it seems particularly important that alternatives to palm oil that are promoted have a historical local tradition.

Rice bran oil is particularly promising in this regard. Our results have shown how sections of industry are already tapping into health perceptions regarding rice bran oil to expand its use as a cooking oil as well as a replacement for palm and other oils in food processing applications. Thailand, of course, is the world’s leading producer of rice and thus rice bran oil has the benefit of being viewed as indigenous. Rice bran oil’s saturated fat content is significantly lower than that of palm oil, while its lower linoleic acid content makes it more oxidatively stable than soyabean oil. Thus rice bran oil holds particular promise as a palm oil substitute in frying applications. Most *et al.* ^(^ ^38^ ^)^ have reported that rice bran oil has total and LDL cholesterol-lowering effects in man and that this is likely due to γ-oryzanol and other unsaponfiables that it contains, rather than its fatty acid structure itself.

However, although rice bran oil has a potential role to play in a strategic diversification of oils in the food system, the role does have limitations, particularly in light of problems related to rice bran sourcing. A strategic plan for policy support, such as in infrastructure development, to ease some of these constraints, would help realize some of this potential. Alternative oils such as rice bran and sunflower oils typically cost at least 30 % more than palm oil at retail outlets. This is a significant price differential for the price-sensitive poor. However, it is important to consider that this price differential may partially reflect differential policy support, including the retail price cap on palm oil. More balanced policy support may result in lower price differentials, making alternative oils more relevant to the poor.

### 2. A strategic long-term rebalancing towards the use of palm oil for biofuels and oleochemicals

Our results have shown that food use of palm oil, particularly its consumption by the poor, is considered the top priority for palm oil by policy makers. Our results further suggest that all other related policy considerations are subjugated to maintain availability and low prices for palm cooking oil. However, it is possible to maintain the viability of the economically important palm oil sector while potentially reaping health benefits by reducing food use. Although food use is prioritized in policy, biofuel use growth has been instrumental to growth of the sector in recent years. Biofuel use of palm oil is already large relative to food use in Thailand, and the petrochemical industry has successfully coped with increases in the biofuel mandates in the past. A range of other oleochemical applications for palm oil is also possible^(^ ^39^ ^)^.

### 3. Harnessing Thailand’s strong research and development capabilities in food technology to design solutions

There is much potential for technological intervention to improve the health profile of edible oils in the Thai food system, replacing partially hydrogenated or highly saturated oils with oils containing a greater proportion of unsaturated fat in a range of food processing applications. Our results have shown how, contrary to the conventional wisdom in food manufacturing that suggests only a narrow range of oils including palm is suitable for deep frying, sections of the food industry have switched from palm to rice bran oil in certain deep frying applications. Such substitution is also being explored in other areas. Skeaff^(^ ^24^ ^)^ discusses a range of lipid technology applications, including interesterification, blending oils, fractionating tropical oils and developing trait-enhanced oils, that may be suitable under different circumstances. Critically, Thailand has given top priority to its food sector as an engine for growth, and has correspondingly invested in and built up strong research and development capabilities in food technology. This capacity, located in the major universities and research institutes, can be harnessed to work with industry in developing solutions. However, this will require policies that incentivize switching of oils and support innovation in this specific area.

A combination of factors makes it unlikely that policies will emerge to restrict the food use of palm oil in Thailand in the medium run. However, as described above, it is feasible to conceive of a long-term strategy of diversification of oils in food use that accommodates existing political and economic interests and builds on Thailand’s strengths and local capacities. Such a strategy may offer future benefits in terms of safeguarding population health and the environment. The challenge lies in convincing policy makers now of the importance of developing such strategic vision for the future.

## References

[1] World Health Organization (2011) Global Status Report on Non-Communicable Diseases. Geneva: WHO.

[2] Misra A, Singhal N & Khurana L (2010) Obesity, the metabolic syndrome, and type 2 diabetes in developing countries: role of dietary fats and oils. J Am Coll Nutr 29, Suppl. 3, 289S–301S. 20823489 10.1080/07315724.2010.10719844

[3] Drewnowski A & Popkin BM (1997) The nutrition transition: new trends in the global diet. Nutr Rev 55, 31–43. 9155216 10.1111/j.1753-4887.1997.tb01593.x

[4] Popkin BM (2002) The shift in stages of the nutrition transition in the developing world differs from past experiences! Public Health Nutr 5, 205–214. 12027286 10.1079/PHN2001295

[5] Li Y, Hruby A, Bernstein AM et al. (2015) Saturated fats compared with unsaturated fats and sources of carbohydrates in relation to risk of coronary heart disease: a prospective cohort study. J Am Coll Cardiol 66, 1538–1548. 26429077 10.1016/j.jacc.2015.07.055 PMC4593072

[6] World Health Organization (2013) *Monitoring Framework and Targets for the Prevention and Control of NCDs: A Comprehensive Global Monitoring Framework, Including Indicators, and a Set of Voluntary Global Targets for the Prevention and Control of Non-Communicable Diseases*. Geneva: WHO.

[7] Popkin BM & Gordon-Larsen P (2004) The nutrition transition: worldwide obesity dynamics and their determinants. Int J Obes Relat Metab Disord 28, Suppl. 3, S2–S9. 15543214 10.1038/sj.ijo.0802804

[8] Byerlee D, Falcon W & Naylor R (2016) *The Tropical Oilseeds* Revolution. New York: Oxford University Press.

[9] Murphy DJ (2014) The future of oil palm as a major global crop: opportunities and challenges. J Oil Palm Res 26, 1–24.

[10] US Department of Agriculture, Foreign Agriculture Service (2016) Palm Oil: World Supply and Distribution. http://apps.fas.usda.gov/psdonline/ (accessed October 2016).

[11] PalmOilWorld.org (2011) Sustainable Palm Oil Developments in Malaysia. http://www.palmoilworld.org/sustainability.html (accessed October 2016).

[12] Sun Y, Neelakantan N, Wu Y et al. (2015) Palm oil consumption increases LDL cholesterol compared with vegetable oils low in saturated fat in a meta-analysis of clinical trials. J Nutr 145, 1549–1558. 25995283 10.3945/jn.115.210575

[13] Fitzherbert E, Struebig M, Morel A et al. (2008) How will oil palm expansion affect biodiversity? Trends Ecol Evol 23, 538–545. 18775582 10.1016/j.tree.2008.06.012

[14] Houghton RA (2005) Tropical deforestation as a source of greenhouse gas emissions. In Tropical Deforestation and Climate Change, pp. 13–21 [P Mouthino and S Schwartzman, editors]. Belem, Brazil and Washington, DC: Amazonian Institute for Environmental Research and Environmental Defense.

[15] Dangour AD, Green R, Häsler B et al. (2012) Linking agriculture and health in low- and middle-income countries: an interdisciplinary research agenda. Proc Nutr Soc 71, 222–228. 22420829 10.1017/S0029665112000213

[16] Dangour AD, Hawkesworth S, Shankar B et al. (2013) Can nutrition be promoted through agriculture-led food price policies? A systematic review. BMJ Open 3, e002937. 10.1136/bmjopen-2013-002937 PMC3696869 23801712

[17] Jensen JD & Smed S (2013) The Danish tax on saturated fat – short run effects on consumption, substitution patterns and consumer prices of fats. Food Policy 42, 18–31.

[18] Uusitalo U, Feskens EJ, Tuomilehto J et al. (1996) Fall in total cholesterol concentration over five years in association with changes in fatty acid composition of cooking oil in Mauritius: cross sectional survey. BMJ 313, 1044–1046. 8898594 10.1136/bmj.313.7064.1044 PMC2352358

[19] Food and Agriculture Organization (2016) FAOSTAT Statistics Database. Rome: FAO.

[20] Kosulwat V (2002) The nutrition and health transition in Thailand. Public Health Nutr 5, 183–189. 12027283 10.1079/PHN2001292

[21] Kunnathum J, Makka N, Aungkulanon S et al. (2009) A comparative risk assessment of health burden attributable to modifiable risk factors in Thailand, 2009: a systematic analysis. Lancet 381, S78.

[22] Institute for Health Metrics and Evaluation (2016) Global Burden of Disease: Thailand. http://www.healthdata.org/thailand (accessed October 2016).

[23] Aekplakorn W, Taneepanichskul S, Kessomboon P et al. (2009) Prevalence of dyslipidemia and management in the Thai population, national health examination survey IV. J Lipids 2014, 249584. 10.1155/2014/249584 PMC3985300 24800083

[24] Skeaff CM (2009) Feasibility of recommending certain replacement or alternative fats. Eur J Clin Nutr 63, Suppl. 2, S34–S49. 19424217 10.1038/sj.ejcn.1602974

[25] Mozaffarian DA & Clarke R (2009) Quantitative effects on cardiovascular risk factors and coronary heart disease risk of replacing partially hydrogenated vegetable oils with other fats and oils. Eur J Clin Nutr 63, Suppl. 2, S22–S33. 19424216 10.1038/sj.ejcn.1602976

[26] Pérez-Ferrer C, Lock K & Rivera JA (2010) Learning from international policies on *trans* fatty acids to reduce cardiovascular disease in low-and middle-income countries, using Mexico as a case study. Health Policy Plan 25, 39–49. 19741052 10.1093/heapol/czp040

[27] Downs SM, Thow AM, Ghosh-Jerath S et al. (2013) From Denmark to Delhi: the multisectoral challenge of regulating *trans* fats in India. Public Health Nutr 16, 2273–2280. 23164094 10.1017/S1368980012004995 PMC10271666

[28] Colón-Ramos U, Lindsay AC, Monge-Rojas R et al. (2007) Translating research into action: a case study on *trans* fatty acid research and nutrition policy in Costa Rica. Health Policy Plan 22, 363–374. 17951318 10.1093/heapol/czm030

[29] Downs SM, Gupta V, Ghosh-Jerath S et al. (2013) Reformulating partially hydrogenated vegetable oils to maximise health gains in India: is it feasible and will it meet consumer demand? BMC Public Health 13, 1139. 24308642 10.1186/1471-2458-13-1139 PMC3878993

[30] De Souza RJ, Mente A, Maroleanu A et al. (2015) Intake of saturated and *trans* unsaturated fatty acids and risk of all cause mortality, cardiovascular disease, and type 2 diabetes: systematic review and meta-analysis of observational studies. BMJ 351, h3978. 26268692 10.1136/bmj.h3978 PMC4532752

[31] Vafeiadou K, Weech M, Altowaijri H et al. (2015) Replacement of saturated with unsaturated fats had no impact on vascular function but beneficial effects on lipid biomarkers, E-selectin, and blood pressure: results from the randomized, controlled Dietary Intervention and VAScular function (DIVAS) study. Am J Clin Nutr 102, 40–48. 26016869 10.3945/ajcn.114.097089

[32] Fattore E, Bosetti C, Brighenti F et al. (2014) Palm oil and blood lipid-related markers of cardiovascular disease: a systematic review and meta-analysis of dietary intervention trials. Am J Clin Nutr 99, 1331–1350. 24717342 10.3945/ajcn.113.081190

[33] Walt G & Gilson L (1994) Reforming the health sector in developing countries: the central role of policy analysis. Health Policy Plan 9, 353–370. 10139469 10.1093/heapol/9.4.353

[34] Ulin PR, Robinson ET & Tolley EE (2005) *Qualitative Methods in Public Health* . San Francisco, CA: JosseyBass.

[35] Eyres L, Eyres MF, Chisholm A et al. (2016) Coconut oil consumption and cardiovascular risk factors in humans. Nutr Rev 74, 267–280. 26946252 10.1093/nutrit/nuw002 PMC4892314

[36] Timmer CP (1989) Food price policy: the rationale for government intervention. Food Policy 14, 17–27.

[37] Rimpeekool W, Seubsman SA, Banwell C et al. (2015) Food and nutrition labelling in Thailand: a long march from subsistence producers to international traders. Food Policy 56, 59–66. 26538793 10.1016/j.foodpol.2015.07.011 PMC4608433

[38] Most MM, Tulley R, Morales S et al. (2005) Rice bran oil, not fiber, lowers cholesterol in humans. Am J Clin Nutr 81, 64–68. 15640461 10.1093/ajcn/81.1.64

[39] Rupilius W & Ahmad S (2007) Palm oil and palm kernel oil as raw materials for basic oleochemicals and biodiesel. Eur J Lipid Sci Technol 109, 433–439.

